# The association of antenatal D-dimer and fibrinogen with postpartum hemorrhage and intrauterine growth restriction in preeclampsia

**DOI:** 10.1186/s12884-021-04082-z

**Published:** 2021-09-05

**Authors:** Hailing Shao, Shichu Gao, Dongru Dai, Xiaomin Zhao, Ying Hua, Huijun Yu

**Affiliations:** 1 Department of Obstetrics and Gynecology, the Second Affiliated Hospital of Wenzhou Medical University, Wenzhou, 325027 China; 2 Department of Obstetrics and Gynecology, Wenzhou People Hospital, Wenzhou, China

**Keywords:** D-dimer, Fibrinogen, Preeclampsia, Postpartum hemorrhage, Intrauterine growth restriction

## Abstract

**Background:**

D-dimer and fibrinogen were verified to be altered in preeclampsia. This study was to evaluate the associations of D-dimer and fibrinogen plasma levels with postpartum hemorrhage or intrauterine growth restriction in preeclamptic women.

**Methods:**

This was a retrospective study that recruited 278 preeclamptic women with singleton pregnancy from January 2016 to December 2019. Patients were allocated into five groups: mild preeclampsia (mPE) (n=68), mild preeclampsia with postpartum hemorrhage (mPE+PPH) (n=13), severe preeclampsia (sPE) (n=112), severe preeclampsia with postpartum hemorrhage (sPE+PPH) (n=17) and severe preeclampsia with intrauterine growth restriction (sPE+IUGR) (n=68). The antenatal D-dimer and fibrinogen plasma levels were analyzed among the groups. Logistic regression was used to determine the correlation between serum indexes and PPH or IUGR in preeclampsia.

**Results:**

The antenatal D-dimer plasma levels were significantly higher in the sPE+PPH group than that in the sPE group (2.02 μg/ml versus 1.37 μg/ml, *P* = 0.001), but there was no difference in fibrinogen. Elevated D-dimer was associated with PPH among severe preeclamptic women (adjusted odds ratio (aOR) [95% CI]: 3.093 [1.527-6.264], *P* = 0.002). No differences in D-dimer and fibrinogen were found between the mPE and mPE+PPH groups or between the sPE and sPE+IUGR groups.

**Conclusions:**

Elevated antenatal plasma D-dimer level may be associated with postpartum hemorrhage in severe preeclampsia, but not with intrauterine growth restriction. Future prospective clinical trials are needed to investigate the predictive value of D-dimer in postpartum hemorrhage in severe preeclampsia.

## Background

Preeclampsia (PE), characterized by new-onset hypertension and proteinuria occurring after 20 weeks of gestational age, is a pregnancy-specific disorder involving multiple organ damage [1]. It affects approximately 4.6% of pregnant women worldwide [2], and is closely associated with adverse pregnancy outcomes, such as intrauterine growth restriction (IUGR) [3], one of the most important causes of death of perinatal infants [4]. In addition, pregnant women with PE are at higher risk of postpartum hemorrhage (PPH) due to uterine atony [5]. Preeclampsia is a dynamic disease and it can present as mild preeclampsia (hypertension with proteinuria only) or develop rapidly into severe preeclampsia (preeclampsia with impaired function of multiple organs and systems), eclampsia (seizures that occur based on pre-eclampsia and cannot be explained by any other cause) or HELLP syndrome (hemolysis, elevated liver enzymes and low platelets), resulting in high fetal and maternal mortality [6]. The timing of diagnosis and treatment depends on the clinical manifestations or laboratory indexes caused by target organ damage, which may miss the optimal intervention time.

A prominent feature reported in PE is the more exacerbated hypercoagulable state, containing platelet activation [7], overproduction of thrombin [8] and alterations of fibrinolytic factors [9]. The activation of platelet may cause thrombosis in terminal vascular branches of target organs. The increased thrombin generation is thought to be associated with endothelial dysfunction, platelet activation and pro-inflammatory cytokines. Additionally, anti-type-1 angiotensin II receptor (AT1) autoantibodies stimulate the release of plasminogen activator inhibitor-1 (PAI-1) [10]. Fibrin deposition was widely found in PE [10], seemingly suggesting that the balance between coagulation and fibrinolysis was broken up [9]. D-dimer is the ultimate degradation product of a fibrin clot cross-linked by factor XIII [11]. Several studies have explored the associations between D-dimer, fibrinogen and mild PE (mPE) or severe PE (sPE), and the possible value of D-dimer and fibrinogen in predicting PE [3, 12–14]. And we notice that these two indicators alter in pregnancies complicated with IUGR or PPH [4, 5].

This study aimed to evaluate the associations of D-dimer and fibrinogen plasma levels with PPH or IUGR in preeclamptic women.

## Methods

**Fig. 1:**
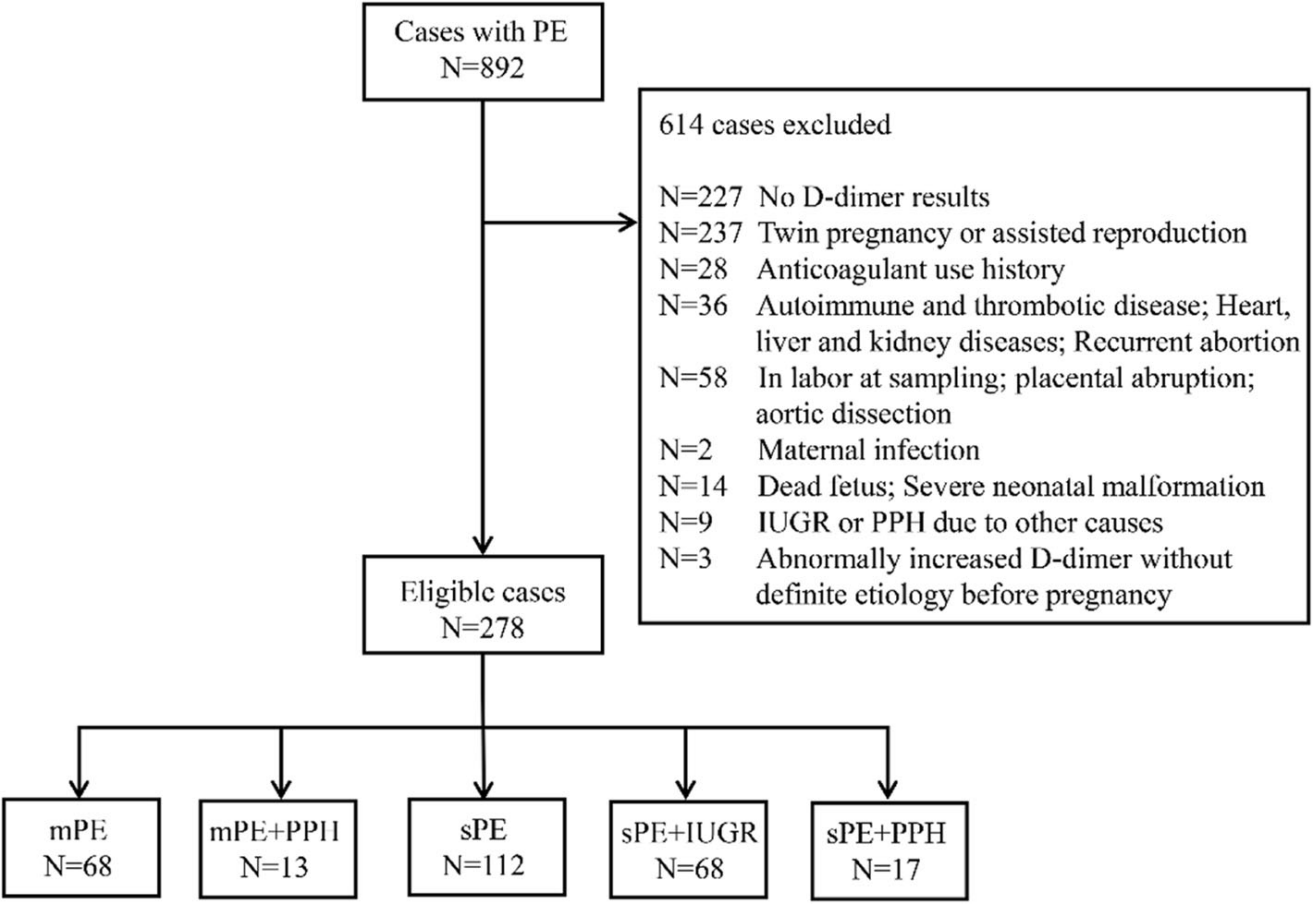
The distribution of cases. NP= normal pregnancy, GW= gestational week, mPE= mild preeclampsia, sPE= severe preeclampsia, PPH= postpartum hemorrhage, IUGR= intrauterine growth restriction

We conducted a retrospective cohort control study in the Second Affiliated Hospital of Wenzhou Medical University from January 2016 to December 2019, including 278 preeclamptic women with singleton pregnancy. This study was approved by the Hospital Research Ethics Committee. All PE patients were hospitalized to give birth at this hospital. As is detailedly showed in Fig. 1, PE patients were categorized into five subgroups according to severity and complications (IUGR and PPH) of this disorder, including the mPE (n=68), mPE+PPH (n=13), sPE (n=112), sPE+PPH (n=17), sPE+IUGR (n= 68) groups. The diagnosis of PE is strictly following International Society for the Study of Hypertension in Pregnancy (ISSHP) guidelines [15] and the American College of Obstetrics and Gynecology (ACOG) guidelines. Cases were considered as severe preeclampsia if they met any of the following criteria: blood pressure ≥160/110 mmHg, thrombocytopenia, maternal organ injury (e. g. liver, kidney), pulmonary edema, new-onset headache or visual symptoms, or uteroplacental dysfunction, such as intrauterine growth restriction. IUGR was defined as the estimated fetal weight was less than the 10th percentile for gestational age and then verified after birth. The PPH was used to describe that the amount of blood loss was more than 500ml within 24 hours after vaginal delivery and more than 1000ml after cesarean section. Pregnant women with immunological diseases, hepatitis, pre-existing renal disease, hematological diseases, malignancy, deep venous thrombosis, recurrent spontaneous abortion, smoking history, anticoagulant drug-use history, assisted reproduction, twin pregnancy, placental abruption, stillbirth, and clinical chorioamnionitis were excluded. Those who were in labor at blood sampling were also excluded.

Clinical data including age, body mass index (BMI), gravidity, parity, gestational age, blood pressure on admission and pregnancy outcomes were collected from the medical record database. Blood samples were collected before delivery or during antenatal care. The tests of D-dimer and fibrinogen quantification were carried out according to the manufacturer’s guidelines (Stago STA-R Evolution, France). D-dimer levels were measured by the immunoturbidimetry assay and fibrinogen levels were measured by the Clauss method. All the measurements were done with the same instrument and methodology.

Statistical analysis was performed using SPSS 25.0 software. The normality was analyzed by the Kolmogorov–Smirnov test. Continuous variables conformed to the normal distribution, were presented as mean ± standard deviation (SD) and analyzed by student t-test or one-way ANOVA with LSD post hoc. Continuous variables that did not conform to the normal distribution, such as D-dimer and fibrinogen, were presented as median and interquartile range and analyzed by Mann-Whitney U test. Categorical variables, presented as percentages (%), were analyzed by Pearson's Chi-square test. We conducted stratified analyses by the gestational age at sampling (< 32 weeks, 32-36 weeks, >36weeks) in comparing D-dimer and fibrinogen between the sPE and sPE+IUGR groups. Logistic regression was used to analyze the relationship between biochemical indexes and PE combined with PPH, adjusted for potential confounding factors, including gestational age at sampling, gestational age at onset, gestational age at delivery, age, BMI, scarred uterus, systolic blood pressure (SBP) and diastolic blood pressure (DBP) at admission. These confounders were selected because they were associated with D-dimer and fibrinogen levels or might influence the incidence of PPH. Hosmer-Lemeshow-Test was used to evaluate the goodness of fit of the model and the *P*-value was 0.377. Furthermore, the receiver operating characteristic curve (ROC) was taken to evaluate their predictive values in PE complicated with PPH. *P*-value <0.05 was considered statistically significant.

## Results

### Subject characteristics

The study flow was shown in Fig. 1. A total of 892 preeclamptic patients were screened according to the inclusion criteria. 614 cases were excluded due to missing D-dimer value, twin pregnancy, assisted reproduction, anticoagulant use history, or other maternal and fetal diseases that influenced D-dimer and fibrinogen plasma levels. Finally, 278 eligible cases were divided into five groups: mPE (n=68), mPE+PPH (n=13), sPE (n=112), sPE+PPH (n=17), sPE+IUGR (n=68). Clinical characteristics including maternal age, gravidity, parity, systolic blood pressure (SBP) and diastolic blood pressure (DBP) at admission were similar between the mPE and mPE+PPH groups, sPE and sPE+PPH groups, sPE and sPE+IUGR groups, respectively (*P* > 0.05). Women who gave birth to neonates with IUGR had lower BMIs (*P* < 0.05). There were no significant differences in gestational age at onset and delivery, neonatal birth weight and 1-5 min Apgar scores between the mPE and mPE+PPH groups (*P* > 0.05). The gestational age at sampling showed no statistical significance between the mPE and mPE+PPH groups (*P* > 0.05), while it was lower in the sPE+IUGR/PPH groups compared with that in the sPE group (*P* < 0.05). Neonatal birthweight and Apgar scores in the sPE+IUGR and sPE+PPH groups were markedly lower than those in the sPE group, which might be related to smaller gestational age in sPE with IUGR/PPH (*P* < 0.05). However, the comparison of stratified gestational age at sampling (< 32 weeks, 32-36 weeks, >36 weeks) between the sPE and sPE+IUGR groups showed no difference (*P* > 0.05) (Table 1).

**Table 1:** Baseline characteristics of the sPE, sPE+PPH and sPE+IUGR groups

	mPE (n=68)	mPE+PPH (n=13)	*P*^a^	sPE (n=112)	sPE+IUGR (n=68)	sPE+PPH (n=17)	*P*^b^	*P*^c^
Age	30.34±5.60	30.77±6.06	0.802	30.94±5.51	31.37±5.18	31.18±4.83	0.605	0.866
BMI(kg/m^2^)	29.69±3.76	29.72±4.30	0.978	28.84±3.64	27.69±3.69	27.15±3.30	0.043	0.075
Gravidity	2 (1,3)	2 (1,4)	0.543	3 (1,4)	3(2,4)	3 (2,4)	0.333	0.546
Parity	0 (0,1)	1 (0,1)	0.188	1 (0,1)	1(0,1)	1 (0,1)	0.853	0.817
Scarred uterus (%)	16 (23.5%)	2 (15.4%)	0.777	37 (33.0%)	23(33.8%)	5 (29.4%)	0.913	0.766
SBP at admission (mmHg)	142.56 ±11.42	146.62±13.07	0.255	163.78±17.58	159.90±20.27	166.24±16.08	0.177	0.588
DBP at admission (mmHg)	89.91±8.96	93.69±11.69	0.189	100.11±12.42	98.66±13.14	103.59±8.89	0.460	0.268
Gestational age at onset (weeks)	37.71 (36.04, 39.39)	37.00 (33.64,40.07)	0.872	33.50 (30.00,36.57)	30.93 (28.14,35.25)	30.00 (27.36,32.50)	0.047	0.027
Gestational age at sampling (weeks)	38.36 (37.32, 39.54)	39.29 (36.57,40.42)	0.414	34.93 (32.18,37.96)	33.71 (31.29,36.57)	30.57 (28.50,35.36)	0.005	0.006
Gestational age at delivery (weeks)	38.86 (37.86, 39.96)	39.71 (37.79,40.71)	0.306	35.57 (32.39,38.14)	33.00 (30.75,36.43)	31.14 (29.14,35.57)	0.004	0.004
< 32				30.43 (28.26,31.21)	30.64 (29.79,31.29)		0.254	
32-36				34.29 (33.32,34.82)	34.14 (32.93,35.00)		0.197	
>36				38.07 (37.14,39.29)	37.36 (36.68,38.89)		0.144	
Cesarean rate (%)	41 (60.3%)	4 (30.8%)	0.050	97 (86.6%)	57 (83.8%)	15 (88.2%)	0.607	1.000
Birth weight(g)	3375 (2900,3737.5)	3480 (3290,3805)	0.222	2570 (1657.5,2995)	1485 (1090,1972.5)	1360 (915,1825)	0.000	0.000
1min Apgar score	10 (9,10)	10 (9,10)	0.182	9 (9,10)	9(8,10)	8 (6,9)	0.020	0.000
5min Apgar score	10 (10,10)	10 (10,10)	0.409	10 (10,10)	10(9,10)	9 (8,10)	0.078	0.000

Data are given as mean ± SD, n (%) or median (interquartile range, IQR)

^a^ The comparison between mPE group and mPE+PPH group

^b^ The comparison between sPE group and sPE+IUGR group

^c^ The comparison between sPE group and sPE+PPH group

### D-dimer and Fibrinogen in PE Complicated with PPH

**Fig. 2:**
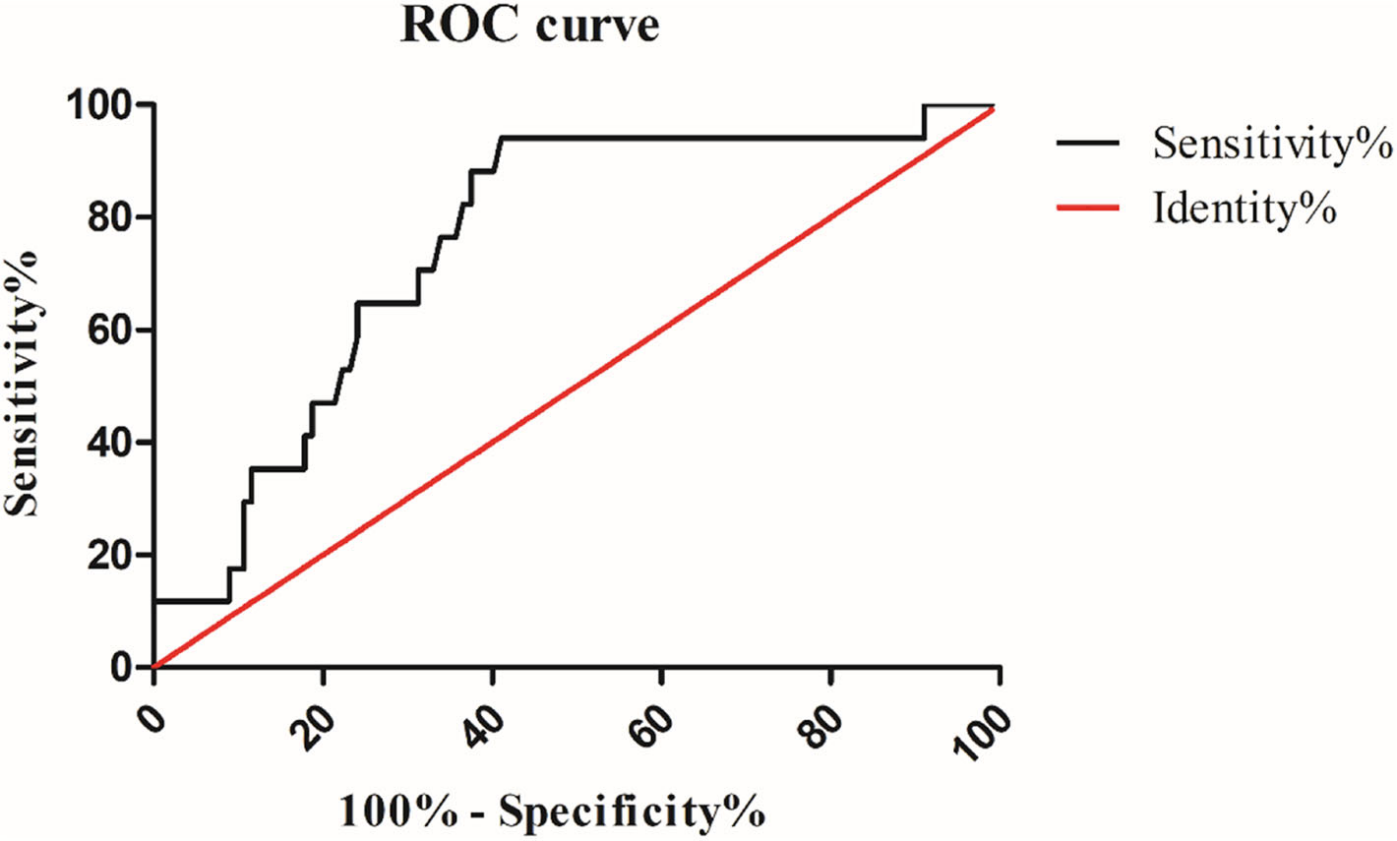
ROC curve of D-dimer

The level of plasma D-dimer in the mPE+PPH group was numerically higher than that in the mPE group, but the difference was not statistically significant (1.17 (0.97, 1.76) versus 1.30 (1.17, 2.33) μg/ml, *P* > 0.05). However, severe preeclamptic women complicated with PPH had observably higher D-dimer plasma levels than those without PPH (2.02 (1.73, 2.50) versus 1.37 (0.94, 1.91) μg/ml, *P* < 0.05). There was no significant difference in the level of plasma fibrinogen between the patients with PPH and the patients without PPH either in mPE or sPE groups (Table 2). Multivariate logistic regression showed that higher D-dimer was associated with PPH in sPE (odds ratio (OR) [95% CI]: 1.991 [1.236-3.208], *P* = 0.005; adjusted odds ratio (aOR) [95% CI]: 3.093 [1.527-6.264], *P* = 0.002), while fibrinogen was independent with PPH (OR [95% CI]: 0.720 [0.420-1.233], *P* = 0.231; aOR [95% CI]: 1.425 [0.682-2.979], *P* = 0.347), after adjusting for gestational age at sampling, gestational age at onset, gestational age at delivery, age, BMI, scarred uterus, SBP at admission, DBP at admission. Furthermore, we estimated the value of D-dimer in predicting PPH in severe preeclamptic women by the ROC analysis. The cut-off point for D-dimer plasma level was 1.555 μg/ml with a sensitivity of 94.1% and a specificity of 58.6% (Fig. 2, Table 3).

**Table 2:** D-dimer and fibrinogen in the PE and PE+PPH groups

	mPE (n=68)	mPE+PPH (n=13)	*P*^a^	sPE (n=112)	sPE+PPH (n=17)	*P*^b^
D-dimer (μg/mL)	1.17 (0.97,1.76)	1.30 (1.17,2.33)	0.124	1.37 (0.94,1.91)	2.02 (1.73,2.50)	0.001
Fibrinogen (g/L)	4.73 (4.31,5.40)	5.13 (4.37,5.31)	0.537	4.52 (3.95,5.22)	3.95 (3.73,5.38)	0.290

Data are given as median (interquartile range, IQR)

^a^ The comparison between mPE group and mPE+PPH group

^b^ The comparison between sPE group and sPE+PPH group

**Table 3:** ROC curve of D-dimer for evaluation of sPE with PPH

Variables	Cut-off value	Sensitivity	Specificity	AUC	*P*	95% CI	
						Lower limit	Upper limit
D-dimer	1.555	94.1%	58.6%	0.752	0.001	0.639	0.865

### D-dimer and Fibrinogen in sPE Complicated with IUGR

The comparisons of D-dimer and fibrinogen were shown in Table 4. In sub-analyses stratified by gestational age at sampling (< 32 weeks, 32-36 weeks, >36 weeks), there were no significant differences in D-dimer and fibrinogen between the sPE group and the sPE+IUGR group (*P* > 0.05).

**Table 4:** D-dimer and fibrinogen in the sPE and sPE+IUGR groups stratified by different gestational age at sampling

`	sPE n=112	sPE+IUGR n=68	*P*
Gestational age < 32 weeks	n=26	n=30	
D-dimer (μg/mL)	1.35(0.81,2.32)	1.24(0.52,1.98)	0.254
Fibrinogen (g/L)	4.01(3.35,4.71)	4.24(3.92,5.06)	0.110
Gestational age ≥32 and ≤ 36weeks	n=36	n=20	
D-dimer (μg/mL)	1.14(0.78,1.71)	1.38(0.87,2.21)	0.197
Fibrinogen (g/L)	4.75(3.98,5.41)	4.14(3.42,5.35)	0.110
Gestational age > 36 weeks	n=50	n=18	
D-dimer (μg/mL)	1.58(1.05,2.09)	1.24(0.98,1.59)	0.144
Fibrinogen (g/L)	4.61(4.18,5.25)	4.16(3.52,4.84)	0.061

Data are given as median (interquartile range, IQR)

## Discussion

Preeclampsia is the second common cause of maternal death worldwide. Preeclampsia-related complications such as PPH and IUGR pose an increased rate of preterm labor and cesarean section [16]. To date, numerous studies focused on finding ideal biomarkers regarding coagulation and fibrinolytic systems, such as D-dimer, fibrinogen, plasminogen activator Inhibitor-1 (PAI-1), tissue-type plasminogen activator (t-PA), for aiding in the early prediction of PE before manifestations occur [9, 13, 17–19]. D-dimer and fibrinogen are easily available laboratory indexes. Thus, in this retrospective study, we chose D-dimer and fibrinogen as the potential indicators to discriminate between preeclampsia with PPH/IUGR and preeclampsia alone. To our knowledge, no similar studies have been reported.

Preeclamptic women are more likely to suffer PPH than low-risk women (10% VS 0.4-1.3%) [20]. Significantly higher D-dimer plasma levels were found in sPE group than those in mPE group [3, 21, 22], suggesting the more aggressively activated coagulation and fibrinolysis systems in severe preeclampsia [17], which might lead to more blood loss in sPE women. The D-dimer plasma level increased gradually with the increase of gestational age during normal pregnancy [5, 21, 23]. Our data showed that the median gestational age at sampling of the sPE group was 34.93 weeks, which was smaller than that in the sPE+PPH group (median: 30.57 weeks), while sPE women with PPH had higher D-dimer plasma level. Logistic regression revealed that D-dimer was associated with the advancing risk of PPH in sPE women. Therefore, the antenatal D-dimer value might be associated with PPH in sPE. The cut-off D-dimer value in our study was 1.555 μg/ml with a sensitivity of 94.1% and a specificity of 58.6%. Endo-Kawamura et al. found that D-dimer > 2.7 μg/ml in late pregnancy approximately doubled the risk of PPH [5]. The difference might be due to disparities in testing methods and subjects.

The results of studies as to the relationship between antenatal fibrinogen plasma levels and PPH were inconsistent. Charbit B et al. reported that no correlation was found between the antenatal fibrinogen plasma levels and PPH [24]. Conversely, Endo-Kawamura et al. found that the antenatal fibrinogen plasma level < 4.0 g/L increased the PPH prevalence rate [5]. Moreover, the cut-off fibrinogen value was 3.3 g/L in women with vaginal delivery but not cesarean section [25]. Pregnant women with preeclampsia are in a hypercoagulant state compared with normal pregnancy [9]. As it progresses to a more severe stage, more changes in coagulation and fibrinolysis indicators occur [17]. Compared with the mPE group, the sPE group showed significantly higher levels of plasma D-dimer and fibrinogen [3]. In our study, no significant difference in antenatal fibrinogen was found between the mPE and mPE+PPH groups or between the sPE and sPE+PPH groups. The conflicting results suggested that antenatal fibrinogen could not predict PPH no matter in women with normal pregnancy or with preeclampsia.

Both preeclampsia and IUGR were classified as the Great Obstetrical Syndromes, because they shared the same pathological mechanisms including inadequate uterine spiral artery remodeling and poor placentation [26]. Higher fibrinogen but not D-dimer in the third trimester was reported in women with IUGR [4]. But among preeclamptic women, those with IUGR did not have higher levels of plasma fibrinogen and D-dimer [19]. Similarly, our team found that the stratified comparisons of D-dimer and fibrinogen between the sPE group and the sPE+IUGR group showed no significant differences. Despite the maternal vascular lesions of PE is more serious than that of IUGR [27], we speculated that the lesions in preeclampsia with IUGR were comparable to those in preeclampsia alone, thus maternal serum indexes were similar between the two conditions.

Of note, this was the first report to describe the alterations of antenatal D-dimer and fibrinogen in preeclamptic women combined with PPH or IUGR. However, a limitation of this study was that D-dimer values were missing in some cases, which might lead to selection bias. Besides, this study used retrospective data and only associations but not causations could be derived. Our data cannot be a proof of D-dimer being predictive of preeclampsia in any form. Large prospective trials are needed to investigate the predictive value of D-dimer in PPH in severe preeclampsia.

In conclusion, antenatal D-dimer level may be associated with PPH in severe preeclamptic women. In clinical practice, for sPE women with high levels of prenatal plasma D-dimer, we need to be aware of the occurrence of PPH.

## Data Availability

The datasets used and/or analysed during the current study available from the corresponding author on reasonable request.

## Abbreviations

PE: Preeclampsia
mPE: Mild preeclampsia
sPE: severe preeclampsia
PPH: Postpartum hemorrhage
IUGR: Intrauterine growth restriction
OR: Odds ratio
CI: Confidence interval
AT1: Anti-type-1 angiotensin II receptor
PAI-1: Plasminogen activator inhibitor-1
ISSHP: International Society for the Study of Hypertension in Pregnancy
ACOG: American College of Obstetrics and Gynecology
BMI: Body mass index
SD: Standard deviation
ROC: Receiver operating characteristic curve
SBP: Systolic blood pressure
DBP: Diastolic blood pressure
t-PA: Tissue-type plasminogen activator
